# Exploring the Disease Experience in Women with PCOS: A Qualitative Content Analysis

**DOI:** 10.3390/healthcare13243243

**Published:** 2025-12-11

**Authors:** Miok Kim, Su Jeong Yi

**Affiliations:** College of Nursing, Dankook University, Cheonan 31116, Republic of Korea; aprilsea@dankook.ac.kr

**Keywords:** polycystic ovary syndrome, menstrual disturbances, qualitative research, infertility, hirsutism

## Abstract

**Highlights:**

**What are the main findings?**

- Korean women with PCOS experience complex psychological, social, and cultural burdens, including stigma, appearance-related distress, and uncertainty regarding fertility.
- Eight interrelated themes illustrate how women reinterpret PCOS, struggle with chronicity, and gradually develop acceptance and self-management strategies within their sociocultural context.

**What are the implications of the main findings?**

- Patient-centered care is needed, including clear explanations, empathetic communication, and accessible educational materials tailored to women’s lived experiences.
- Public health strategies should strengthen social awareness, early education, and culturally sensitive support systems to enhance the well-being and self-efficacy of women with PCOS.

**Abstract:**

**Purpose:** This study explored the lived experiences of Korean women with polycystic ovary syndrome (PCOS) and examined how cultural expectations surrounding femininity and reproductive roles shaped their illness experiences. **Methods:** In-depth interviews were conducted with fifteen women diagnosed with PCOS at a women’s hospital in South Korea. Data were analyzed using qualitative content analysis. **Results:** Eight themes emerged: (1) A Disease That Doesn’t Feel Like a Disease: Ambiguity and Reinterpretation; (2) Isolation of Body and Mind Amid Stigma and Misunderstanding; (3) Daily Life Limitations Caused by Visible Symptoms; (4) Ambivalent Feelings Surrounding Pregnancy; (5) Difficulties in Self-Management Due to Lack of Information; (6) Psychological Exhaustion From Chronicity and Lack of Control; (7) Awareness and Practices for Living With the Condition; and (8) Moving Toward a Patient-Centered Healthcare Environment. Participants experienced emotional distress related to unpredictable menstrual cycles, infertility fears, appearance concerns, and social misunderstanding. They also reported insufficient communication during clinical encounters. Cultural norms rooted in Confucian values regarding appearance and reproductive responsibility significantly exacerbated these challenges, influencing emotional distress, coping strategies, and healthcare interactions. **Conclusions**: Women with PCOS experience complex psychological, social, and practical challenges that extend beyond physical symptoms. Culturally sensitive, patient-centered approaches—along with improved information delivery and public awareness—are essential to support their well-being, self-efficacy, and long-term disease management.

## 1. Introduction

Polycystic ovary syndrome (PCOS) is one of the most common endocrine disorders among women of reproductive age, with a global prevalence ranging from 4% to 20% depending on the diagnostic criteria used [1]. In South Korea, the number of women seeking clinical care for PCOS has increased nearly threefold over the past decade, suggesting growing awareness as well as a rising clinical burden [2]. Clinically, PCOS presents with various symptoms—including menstrual irregularities, hyperandrogenism, and infertility—and is associated with metabolic complications such as insulin resistance, type 2 diabetes, dyslipidemia, and cardiovascular disease [3,4,5]. These physical and metabolic issues are accompanied by significant psychological burdens. Many studies have reported higher risks of depression, anxiety, and negative body image among women with PCOS, all of which contribute to diminished quality of life [6,7,8,9].

Although PCOS requires long-term management, many women encounter fragmented information, inconsistent explanations, and delayed diagnoses during clinical encounters [10,11,12]. These unmet informational and emotional needs can increase psychological distress, reduce treatment adherence, and impede lifestyle modification, cumulatively worsening health-related quality of life [13,14,15]. Despite these challenges, only a small proportion of affected women receive structured psychological support or counseling [12], highlighting persistent gaps in holistic, patient-centered care.

While qualitative studies have been conducted internationally to examine the psychosocial dimensions of PCOS [10,16,17], research in the Korean context remains limited. South Korean society is influenced by Confucian values, which place substantial expectations on femininity, physical appearance, and reproductive responsibility. These cultural norms may uniquely frame how Korean women interpret menstrual irregularities, infertility risk, weight changes, or visible symptoms such as hirsutism. Consequently, their psychological responses, coping strategies, and healthcare-seeking behaviors may differ from those of women in Western contexts. Understanding these culturally embedded experiences is essential for developing effective nursing and psychosocial interventions.

To examine these experiences in depth, this study employed qualitative content analysis (QCA), which allows systematic exploration of shared patterns and individual variations in illness perception, coping, and meaning making [18,19]. To further enhance interpretive depth, this study adopted Bury’s concept of “biographical disruption” [20]. This framework suggests that chronic illness disrupts life trajectories and challenges fundamental assumptions about health and identity, requiring individuals to adjust and reconstruct meaning. Applying this lens allows PCOS to be understood not only as a medical condition, but also as a disruption to women’s biographies, identities, and social roles within the specific sociocultural context of South Korea.

## 2. Materials and Methods

### 2.1. Study Design

This study employed qualitative content analysis to identify the major problems experienced by women with PCOS and to derive specific implications for nursing interventions.

### 2.2. Participants and Setting

Participants were recruited through purposive sampling from D Women’s Hospital, a specialized women’s healthcare institution located in Gyeonggi-do, South Korea. The hospital is a government-designated fertility center that provides comprehensive reproductive endocrinology services, including infertility treatment, ovulation induction, and management of high-risk pregnancies. In addition to fertility care, the hospital offers a broad range of gynecological services across the female life course, allowing for the recruitment of women with diverse clinical experiences related to PCOS.

The participants of this study were 15 women aged 19–45 years who had been diagnosed with PCOS based on the Rotterdam criteria [21] and were receiving treatment and management at a fertility specialty hospital. All participants were able to engage in interviews and voluntarily agreed to participate in this study. The sample size was primarily determined based on data saturation. After 11 interviews, data began to show repetition, and by the 15th interview, no new information emerged. Data collection was concluded when saturation was reached.

In addition to saturation, the sample size was also considered in relation to methodological recommendations, such as those of Creswell [22]. Creswell suggests that while sample sizes vary by approach (e.g., 20–30 for grounded theory, and around 10 for phenomenology), the duration and quality of data collection are often more critical than the sheer number of participants. Furthermore, previous qualitative studies conducted in Korea were reviewed for reference. Jeon and Kim [23] conducted a study on the experiences of women undergoing infertility treatments with 15 participants, and Kim and Shin [24] explored the illness experiences of young women with gynecologic cancer using 14 participants. Considering these precedents and the wide variation in the diagnosis, symptoms, and management of PCOS [25], this study recruited a total of 15 participants to ensure sufficient depth for qualitative content analysis.

### 2.3. Data Collection

Data were collected from 4 March to 10 May 2024. Each participant took part in one or two interviews, with a duration ranging from 30 min to 1 h and 40 min (average: 1 h). In-depth, face-to-face interviews were conducted at locations convenient for participants, such as near the hospital or in a quiet café. Prior contact was made to establish rapport, and during the interviews, a comfortable and private environment was ensured. The researcher adjusted the pace of the interviews and allowed breaks as needed.

The interview began with a broad, open-ended question: “What was your experience of being diagnosed with PCOS?” Participants were encouraged to discuss their diagnosis and disease management experiences freely. Additional probing questions were used only when necessary to clarify details or encourage elaboration on specific topics, allowing the participants to lead the narrative.

With participants’ consent, all interviews were audio-recorded, transcribed verbatim, and supplemented with field notes describing behaviors, tone, and facial expressions. All data were securely stored on a password-protected personal computer, and files were labeled with codes such as “Participant 1” to ensure confidentiality.

### 2.4. Data Analysis

Data were analyzed using the qualitative content analysis procedure proposed by Krippendorff [19], which consists of three steps.

First, the researchers thoroughly reviewed and repeatedly read the transcripts to gain an in-depth understanding of the text. To enhance reliability, at least two researchers independently analyzed the data and compared results for agreement. Any disagreements in coding were resolved through iterative discussion until consensus was reached, and when needed, a third qualitative expert was consulted to ensure dependability. Second, meaningful statements related to the research topic were identified, grouped, and labeled. The coding process included identifying raw meaning units, condensing their content, and generating initial codes. An example of this analytic progression—from meaning units to codes, sub-themes, and themes—is presented in Table 1. Third, the data were conceptualized and categorized by reorganizing related content based on similarities, relationships, and commonalities. Throughout the analysis, reflexive memos and analytic journals were maintained to ensure transparency and trace the interpretive process from descriptive to theoretical levels. This process was repeated interactively to refine understanding and confirm the appropriateness of the emerging categories while preserving the participants’ voices and contexts.

### 2.5. Rigor

The rigor of this study was ensured based on the four criteria by Lincoln and Guba [26].

Credibility: In-depth interviews centered on open-ended questions were conducted to encourage participants’ authentic narratives, and all interviews were recorded and transcribed verbatim. Participants’ own words were used during coding, and categories were named to reflect the full scope of the data.

Transferability: Theoretical sampling was applied to recruit participants with diverse characteristics (e.g., age, occupation, religion, timing of PCOS diagnosis, and infertility experience). Data collection and analysis continued until saturation. The findings were later reviewed by two women with PCOS who met the inclusion criteria but were not original participants, confirming that their experiences aligned with the study results.

Dependability: The first author directly conducted all stages of data collection and analysis and clearly documented each step. Additionally, feedback was sought from a nursing faculty member experienced in qualitative research to ensure the validity of the themes and results.

Confirmability: Member checking was performed by sharing the findings and theoretical framework with participants. During interviews, the researcher verified interpretations with participants to avoid imposing personal biases on the analysis.

### 2.6. Researcher Preparation and Reflexivity

The primary researcher was a clinical nurse with extensive experience in infertility care and has conducted several studies addressing the psychological and physical challenges faced by women with infertility. The co-researcher holds a doctoral degree in qualitative research and has explored women’s experiences with infertility and reproductive health.

During the study design and data analysis phases, both researchers collaboratively discussed topic selection, developed interview questions, and agreed on the analytical framework. They independently coded the transcripts and later compared and refined their analyses through regular discussions to ensure consistency and analytical rigor.

Throughout the research process, the researchers remained reflexively aware of their preconceptions regarding PCOS as a chronic and potentially stigmatized condition. To minimize bias, they maintained reflexive journals and analytical memos documenting interpretive decisions and data patterns. Interpretations were continually compared with the raw data, and peer debriefing sessions were conducted. Additionally, an experienced qualitative researcher provided external feedback on theme development to enhance neutrality and credibility.

### 2.7. Ethical Considerations

This study was approved by the Institutional Review Board of D University (IRB no. 2023-08-041-003). All participants received a thorough explanation of this study’s purpose, procedures, potential benefits and risks, anonymity, voluntary participation, and the right to withdraw at any time. Written informed consent was obtained prior to participation.

All data were anonymized and securely stored on encrypted devices. No personally identifiable information was collected. Participants were informed that they could discontinue or withdraw from the interview at any point and were provided with information about professional counseling services if needed. The data were used solely for research purposes and will be reported anonymously in academic publications and presentations.

### 2.8. Translation Procedure

All interviews were conducted in Korean and transcribed verbatim. To ensure linguistic and cultural accuracy, the transcripts were first translated into English by a bilingual researcher and then back-translated into Korean by another bilingual expert. The original and back-translated versions were compared to confirm meaning equivalence. Discrepancies were discussed until consensus was reached, with particular attention to preserving participants’ emotional tone and idiomatic expressions. The quotations presented in this paper, therefore, reflect meaning equivalence rather than literal translation, maintaining participants’ voices as authentically as possible.

## 3. Results

The participants of this study comprised 15 women who had been diagnosed with PCOS and were receiving clinical management. The mean age was 32.2 ± 4.09 years, with three participants in their 20s and twelve in their 30s. Two participants were unmarried, and thirteen were married; two had children, while thirteen did not. The mean age at PCOS diagnosis was 23.4 ± 5.37 years, and the average disease duration to date was 8.5 ± 4.02 years (see Table 2).

The illness experiences of women diagnosed with PCOS were categorized into eight main themes: (1) A Disease That Doesn’t Feel Like a Disease: Ambiguity and Reinterpretation, (2) Isolation of Body and Mind Amid Stigma and Misunderstanding, (3) Daily Life Limitations Caused by Visible Symptoms, (4) Ambivalent Feelings Surrounding Pregnancy, (5) Difficulties in Self-Management Due to Lack of Information, (6) Psychological Exhaustion from Chronicity and Lack of Control, (7) Awareness and Practices for Living with the Condition, and (8) Moving Toward a Patient-Centered Healthcare Environment. These experiences appeared to emerge in a complex manner, shaped by the chronic nature of PCOS, its reproductive implications, and the cultural and social context of Korean society (see Table 3).

### 3.1. A Disease That Doesn’t Feel Like a Disease: Ambiguity and Reinterpretation

Participants experienced considerable shock and frustration following a PCOS diagnosis, yet they often did not perceive it as a clear-cut disease, accepting it instead as a vague or ambiguous condition. The initial shock functioned as a moment of biographical disruption, abruptly challenging their previous assumptions about their bodies and future fertility. For some, the disease felt invisible or insignificant because symptoms caused little discomfort, weakening their sense of urgency and leading them to downplay the condition. Others cognitively reframed the absence of menstruation or symptoms as convenient, minimizing the seriousness of PCOS in everyday life. Overall, women moved between shock, uncertainty, and reinterpretation as they tried to make sense of the diagnosis and understand what PCOS meant for their bodies and future reproductive health.

#### 3.1.1. Shock and Confusion at Diagnosis

Upon receiving a PCOS diagnosis, participants experienced unexpected and intense shock. For those who had not previously perceived any issues with their bodies, the diagnosis was akin to an unexpected reality comparable to a cancer diagnosis. This reaction extended beyond mere surprise, encompassing anxiety about future fertility and long-term health concerns. This initial shock may be understood as the first moment of biographical disruption, as the unexpected diagnosis challenges women’s prior assumptions about their health and introduces a sudden break from their anticipated sense of bodily normalcy.

*“I was so shocked, it felt like being given a cancer diagnosis.”*

(Participant 4)

*“I felt really frustrated at that time… It’s a women’s health condition, hard to talk about, hard to manage… Later, I searched online and found it’s very tricky to manage, with no concept of complete cure… menstruation wasn’t normal while others had it normally… (omitted)… Later, when I thought about getting married, I realized I might have trouble conceiving. Overall, I felt very depressed and stressed.”*

(Participant 1)

#### 3.1.2. An Invisible Disease, Unfelt Risk

Participants often viewed PCOS as a limited reproductive issue, recognizing its significance only when preparing for pregnancy. Because it did not cause immediate discomfort or pain, they sometimes failed to perceive its seriousness as a disease. Many participants were unfamiliar with PCOS, lacking a clear understanding of its causes or symptoms, and did not always recognize it as a disease. This invisibility of symptoms may contribute to an early ambiguity within biographical disruption, delaying women’s recognition that their health and life trajectory have begun to shift.

*“I don’t think I really perceived it as a disease. I never thought I had to undergo treatment.”*

(Participant 6)

*“My periods didn’t occur for about three months, so I went to the hospital and was told I had polycystic ovary syndrome. But it didn’t feel like something serious, like diabetes or high blood pressure… it was just something I had, nothing more than that.”*

(Participant 7)

#### 3.1.3. Reinterpretation as Convenience

Some participants perceived the absence of menstruation or menstrual symptoms as a convenience in their current lives, minimizing the negative implications of the disease. With reduced discomfort from periods and no immediate plans for pregnancy, cognitive reinterpretation framed the condition as more convenient than burdensome. This reinterpretation may be understood as an early effort to maintain a sense of normalcy in the face of biographical disruption, as women made sense of their diagnosis by framing the condition as manageable or not requiring immediate concern within the context of their daily lives.

*“As a child, I thought, not having periods is convenient, not being pregnant is convenient… so I just neglected it…”*

(Participant 2)

*“Since I didn’t have any menstrual symptoms, it was actually convenient. And at that time, I didn’t have strong intentions to have children, so it felt fine just the way it was…”*

(Participant 9)

### 3.2. Isolation of Body and Mind Amid Stigma and Misunderstanding

Participants experienced a disruption in their social and personal lives due to PCOS. The condition’s association with reproductive function led to fear of negative judgment and stigma, particularly among unmarried women, who reported discomfort even in visiting a gynecologist. Variability in symptoms and treatment responses among individuals with PCOS further hindered mutual understanding, intensifying feelings of isolation. As a result, participants engaged in strategies such as avoiding disclosure, withdrawing during gynecological visits, and limiting peer interactions, reflecting the broader impact of the illness on their daily lives and social identity.

#### 3.2.1. Social Perceptions That Discourage Disclosure

Participants hesitated to disclose their PCOS to family, partners, friends, or colleagues. Experiences such as taking oral contraceptives, weight changes, or gynecological visits could be misunderstood or interpreted sexually by others, making disclosure difficult. This reluctance to disclose can be understood as a form of careful information control, through which women attempt to protect their moral and social identity from potential stigma, aligning with how biographical disruption can extend into the social realm.

*“I wondered how I could tell my boyfriend… (omitted)… Because it’s birth control, my father might wonder why I’m taking it. Explaining this was difficult and stressful.”*

(Participant 1)

*“My younger sister also has PCOS, and she says it’s embarrassing to talk about it outside because others might hear. I think it would be good if we could change that perception, talk openly, and share information.”*

(Participant 15)

#### 3.2.2. Emotional Discomfort During Gynecological Visits Amid Social Perceptions

Participants perceived society as holding negative views toward unmarried women visiting gynecologists. Even when attending simply for medical care, they felt self-conscious and withdrew emotionally. This emotional withdrawal may illustrate how the biographical disruption of PCOS extends into clinical spaces, where concerns about social judgment deepen women’s sense of discomfort and separation from perceived norms.

*“Many people view visiting a gynecologist negatively, and going alone is also embarrassing…”*

(Participant 15)

*“When women come with a full belly or with their husbands… I sit there alone, feeling awkward. I wonder why I’m here—I’m not preparing for pregnancy, I’m not with anyone, just sitting awkwardly alone…”*

(Participant 1)

#### 3.2.3. Feelings of Being Alone Within the Disease

Although PCOS is a shared diagnosis, participants noted that individual symptoms, reactions, and life contexts differed, making deep emotional exchanges difficult. They experienced relative isolation even in peer relationships. This sense of isolation may reflect the individualized nature of biographical disruption in PCOS, where differing symptom patterns and treatment experiences limit the possibility of shared understanding among peers.

*“Even though we have the same condition, everyone is different, so there’s a sense of loneliness. My pharmacist friend and another friend improved after taking [medication], but it didn’t work for me. Conversations only go so far… even if we share more deeply, there’s no concrete solution. Subtly, situations like this caused a lot of frustration.”*

(Participant 9)

### 3.3. Daily Life Limitations Caused by Visible Symptoms

Participants experienced both physical and psychosocial disruption due to PCOS-related symptoms, including increased body hair, skin changes, and irregular menstruation. These manifestations generated appearance-related stress, heightened sensitivity to social scrutiny, and lifestyle limitations, highlighting that the impact extended beyond physiological effects to disrupt daily life and social functioning.

#### 3.3.1. Confusion and Embarrassment Due to Appearance Changes

Participants reported difficulty in clearly explaining changes in their appearance, such as body hair growth. These changes were inconsistent and recurrent, sometimes appearing and disappearing suddenly, making them hard to control or predict. Often, these changes were noticed through others’ comments, causing embarrassment and shock, and increasing pressure for appearance management. The unpredictable and visible nature of these symptoms may be understood as intensifying biographical disruption, as the loss of control over bodily changes challenges women’s efforts to maintain a stable and socially acceptable self-presentation.

*“Even though I’m experiencing it, I don’t really know what’s happening. It’s hard and changes every day. Sometimes the hair turns red and thick, then disappears… It comes out all of a sudden, it’s chaotic… I just don’t know.”*

(Participant 9)

*“During college, I skipped upper lip hair removal for a while, and a friend said, ‘You should shave it now.’ That comment shocked me so much that I went straight to remove it.”*

(Participant 15)

#### 3.3.2. Anxiety and Lifestyle Limitations Due to Unpredictable Menstruation

Irregular cycles and sudden breakthrough bleeding disrupted participants’ daily plans and caused ongoing stress in activities such as going out, sleeping, and hygiene management. Anxiety persisted even when menstruation was absent, and the uncertainty of its onset threatened emotional stability. This unpredictability may be understood as contributing to biographical disruption by unsettling the temporal rhythm of daily life, requiring women to remain watchful and adapt continually to the possibility of unexpected bleeding.

*“Even in midsummer, breakthrough bleeding kept happening, so for two weeks I had to constantly wear panty liners, which was very uncomfortable. Because I never knew when my period would come, I often had inconvenient experiences when going out. Even as an adult, if it starts while I’m sleeping, it stains sheets and clothes, and I can’t respond immediately, which is very stressful.”*

(Participant 15)

*“When my period is supposed to start but gets delayed, that frustration… the unpredictability… not knowing when it will happen is the most anxiety-inducing, especially when the flow is heavy. The uncertainty constantly causes stress.”*

(Participant 1)

### 3.4. Ambivalent Feelings Surrounding Pregnancy

Participants reported emotional anxiety and biographical disruption stemming from uncertainty about fertility. Irregular menstrual cycles and unpredictable ovulation created concerns about unintended pregnancy, while the chronic nature of PCOS produced vague fears regarding potential infertility. These ambivalent feelings regarding reproductive function influenced women’s identity, life planning, and overall psychological well-being, reflecting the profound impact of the condition on personal biography.

#### 3.4.1. Anxiety About Unpredictable Fertility

Some participants felt anxious about the possibility of unintended pregnancy due to uncertainty in ovulation and contraceptive use. Irregular cycles and difficulty predicting ovulation reinforced this cautious mindset. This anxiety may be understood as reflecting how the unpredictability of the body contributes to biographical disruption, prompting women to remain attentive to the possibility of unintended changes in their reproductive potential.

*“What if I suddenly ovulated and got pregnant? You can’t test every time. Even now, part of me worries, ‘It could happen,’ so I try to be careful just in case.”*

(Participant 9)

#### 3.4.2. Fear of Infertility

Participants expressed anxiety and fear regarding the possibility of difficulty conceiving after a PCOS diagnosis. Information from the internet or negative experiences of others amplified these fears, sometimes leading to concerns about loss of fertility and threats to femininity. This fear may be understood as reflecting a disruption to participants’ envisioned future biography, as uncertainty about fertility introduces instability into their expectations for future life planning. Such fears also intersect with broader social expectations around marriage and childbearing, which may intensify women’s sense of uncertainty about their reproductive future.

*“After hearing about PCOS, I searched online and found that it can lower egg quality… there’s talk about empty follicles and other issues… hearing that my eggs might not be viable made me really scared.”*

(Participant 10)

*“Someone I know said, ‘Sister, you have PCOS too? What will we do… I can’t have children now because of PCOS…’ and started crying. I also have PCOS, so then I thought, I might not be able to have a baby either. This was terrifying.”*

(Participant 12)

### 3.5. Difficulties in Self-Management Due to Lack of Information

Participants experienced decision-making burdens and emotional distress due to insufficient or ambiguous explanations from healthcare providers regarding PCOS. They had to make independent choices about treatment discontinuation, medication use, and fertility potential because clear guidance was not provided. Ambiguous instructions and downplaying of symptoms by providers led to confusion, underestimation of the seriousness of the condition, and delays in active management, reflecting the challenges women face in attempting to interpret and respond to their illness.

#### 3.5.1. Patient Burden in Decision Making Due to Ambiguous Explanations from Healthcare Providers

Some participants had to make decisions on their own regarding the end of treatment, medication discontinuation, fertility potential, and risk of complications because clear guidance was not provided. Such vague instructions led them to underestimate the seriousness of the condition or delay active management. This ambiguity may be understood as complicating the early processes of meaning making and coping work, as women were left to interpret fragmented information and make consequential decisions about their health with limited professional direction.

*“I asked if I could stop taking the pill once my cycle was regulated, but the doctors didn’t know for sure. They said that since everyone’s body and situation is different, they couldn’t give a definitive recommendation. They just said, ‘Once your cycle is regular, you can try stopping it once,’ so that’s what I did.”*

(Participant 4)

*“They just told me, ‘You’re 20, so when it’s time to get pregnant, you can take follicle-stimulating injections,’ so I thought it wasn’t a big deal. If they had told me that leaving it untreated could lead to infertility, maybe I would have put in more effort.”*

(Participant 2)

#### 3.5.2. Negative Emotional Experiences Due to Healthcare Providers’ Responses to Symptoms

Participants reported emotional distress when healthcare providers downplayed the significance of the condition or focused solely on fertility. Such interactions may be perceived as invalidating women’s illness experiences and may complicate their efforts toward early meaning-making and coping work, as they attempt to make sense of their symptoms in the context of explanations that feel incomplete or dismissive.

*“Not having my period meant that my body wasn’t healthy, so I went to the OB-GYN to get the pill to try to regulate it. But one doctor asked, ‘It’s convenient that you don’t have your period, right? You’re not trying to get pregnant, so why care?’ That really hurt me.”*

(Participant 9)

*“They just told me not to worry… that if I wanted to have a baby after getting married, I could do ovulation induction. They said the pill was like a vitamin for someone like me… which made me feel dismissed.”*

(Participant 11)

### 3.6. Psychological Exhaustion from Chronicity and Lack of Control

Participants reported emotional exhaustion, frustration, helplessness, resignation, and regret due to the chronic nature of PCOS and the repetitive processes of disease management. Although they recognized the importance of treatment, the lack of perceptible improvement often led to emotional fatigue. Delayed awareness of the need for early intervention was accompanied by self-blame and regret, illustrating the psychological burden associated with sustaining coping efforts over time.

#### 3.6.1. Frustration and Helplessness During Ongoing Management

Participants described feelings of emotional fatigue and exhaustion when symptoms persisted despite continuous medication and management. The repetitive nature of self-care was perceived as burdensome and, at times, evoked negative reflections about their bodies or identities. These persistent struggles may illustrate the difficulty of meaning-making and coping work in the context of a chronic condition, as the unpredictable course of PCOS disrupts women’s efforts to stabilize their routines and regain a sense of continuity in daily life.

*“Taking medicine every day like my parents do is so sad. I wonder how long I have to live like this…”*

(Participant 4)

*“Carrying this with me all the time feels so heavy, like a burden… Sometimes I want to ignore it, or think that if I just ignore not having my period for a few months, it will be fine. I ask myself, why was I born a woman to go through this? It often feels overwhelming.”*

(Participant 1)

#### 3.6.2. Resignation and Surrender When Efforts Yield No Change

Despite active efforts such as exercise and dietary modifications, participants sometimes felt unable to control the disease. This sense of lack of control gradually led to decreased motivation and resignation. These responses may reflect a point at which ongoing biographical work becomes increasingly difficult, as repeated attempts to regain bodily control do not yield the desired results, making sustained engagement in self-management harder to maintain.

*“It seems like normal people achieve results with this level of effort, but I don’t. After trying for four years, I realize this isn’t something easily resolved… I increasingly feel a sense of surrender rather than determination to overcome it.”*

(Participant 1)

*“I tried various workouts, including CrossFit, but I never felt any improvement. I ended up thinking this is just unavoidable, and I gradually gave up.”*

(Participant 8)

#### 3.6.3. Self-Blame from Belated Awareness

Participants reflected on the time that had passed without sufficient awareness of their condition and expressed regret and self-blame. They repeatedly thought about how earlier management might have alleviated their emotional and physical burden. This retrospective regret may represent an attempt to make sense of the biographical disruption by interpreting the delayed recognition of symptoms as a personal shortcoming, highlighting how women retrospectively negotiate responsibility for their health trajectory.

*“I thought I was too young, born in ’87, to develop diabetes… but now I realize it could have been related. If I had known earlier, maybe I could have taken some action.”*

(Participant 9)

*“I wonder why I didn’t manage it from a young age. If I had, I might not have gone through so much emotional distress. I keep thinking I should have managed it better.”*

(Participant 13)

### 3.7. Awareness and Practices for Living with the Condition

Participants demonstrated acceptance of PCOS as a chronic condition and took responsibility for self-management. Over time, they integrated the condition into their identity and sought strategies to coexist with it in daily life. Recognizing the potential for complications broadened their perspective on overall health, and they actively engaged in self-management through online information-seeking and collaboration with family and supporters, reflecting the process of biographical reconstruction in which women develop new narratives and practices to incorporate PCOS into their lives.

#### 3.7.1. Self-Acceptance

Participants expressed acceptance of PCOS as part of themselves, rather than denying or hiding it. They acknowledged negative aspects, such as physical changes or menstrual irregularities, without attempting to eliminate them entirely, fostering an attitude of embracing their body as it is. This shift in perspective may be understood as part of biographical reconstruction, as women gradually work to integrate the condition into their sense of self and find ways to live with the ongoing changes it brings.

*“When I was younger, I disliked hirsutism… but over time, I started thinking, ‘So what if it’s there?’ It’s my body after all… I just accept myself and live with it.”*

(Participant 2)

*“I decided to focus on what I can do to live well with this condition.”*

(Participant 1)

#### 3.7.2. Redefining the Condition Through Awareness of Complications

Participants recognized PCOS as a health issue beyond appearance, and the potential for complications such as diabetes or metabolic syndrome motivated concrete management behaviors. This realization may represent an important step in biographical reconstruction, as women begin to shift from managing immediate symptoms to adopting a longer-term perspective on maintaining their overall health.

*“Even if hair becomes thicker, I can remove it. But diabetes isn’t something you can manage so easily. I’ve seen my grandmother struggle with diabetes… my mother-in-law knows someone who even had a toe amputated. It made me realize this isn’t a trivial condition.”*

(Participant 9)

*“I didn’t know it was related to diabetes at first. Now that I know, I realize I need to manage it.”*

(Participant 6)

#### 3.7.3. Proactive Information-Seeking for Condition Understanding

Participants actively sought PCOS-related information through online communities, YouTube, and acquaintances. This proactive information seeking may be understood as part of biographical reconstruction, as women turn to external resources to better understand their condition and identify strategies for managing it in daily life.

*“The infertility forums were run by people who really knew their stuff, so I looked at those a lot.”*

(Participant 1)

*“I searched on YouTube and asked questions on knowledge-sharing platforms. I realized I’m not the only one with PCOS, and I shared information about helpful foods with friends who have it too.”*

(Participant 8)

#### 3.7.4. Lifestyle Changes and Family Support

Participants pursued healthier lifestyle habits, such as diet modification, regular exercise, smoking cessation, and inositol supplementation. Some engaged family members in these practices, maintaining motivation through shared efforts. These practical adjustments and the involvement of support systems may be understood as contributing to biographical reconstruction, as shared management provides encouragement and helps women sustain their efforts to adapt to the ongoing demands of the condition.

*“I try to minimize stress because it can exacerbate PCOS… I also take inositol and other recommended supplements.”*

(Participant 5)

*“My mother helps by giving advice and guiding my diet. My twin sister also said, ‘If you need to diet, I’ll join you,’ so we exercise together, which helps me stay motivated.”*

(Participant 1)

### 3.8. Moving Toward a Patient-Centered Healthcare Environment

Participants identified gaps in information provision, communication, and educational systems during PCOS management and expressed a desire for a shift toward a patient-centered environment. Despite PCOS being common, they noted limited social awareness, insufficient public campaigns, and inconsistent explanations from healthcare providers. They hoped for accessible educational materials, opportunities to share experiences, and environments that facilitate peer-to-peer communication, highlighting efforts to reconstruct their biography by seeking supportive structures and integrating social resources into their illness experience.

#### 3.8.1. Need for Education and Awareness Improvement

Participants emphasized the lack of social awareness and educational opportunities regarding PCOS. Compared with other conditions, there is less promotion, prevention-focused education, and open discussion, particularly regarding early recognition of menstrual irregularities after puberty and the creation of an open environment for dialogue. These concerns may suggest a need for broader public awareness efforts, as a more informed and open social environment could support women in navigating their condition and engaging in the ongoing process of biographical reconstruction.

*“For other diseases, there seem to be a lot of campaigns about symptoms and health management, but since PCOS only occurs in women, people seem reluctant to talk about it. Because it only affects women, I feel like less investment is made in prevention, and overall, there seems to be very little education about it.”*

(Participant 12)

*“Although irregular cycles can occur right after menarche, if they continue into later adolescence, it’s important to recognize it as abnormal. I hope there could be a more open environment where we can share, ‘This is my situation, how about yours?’ and go to gynecologists and receive treatment or management without hesitation.”*

(Participant 15)

#### 3.8.2. Expectations for Healthcare Providers’ Explanation, Empathy, and Guidance

Participants expressed that healthcare providers should explain the causes, management strategies, and prognosis of PCOS while acknowledging patients’ anxiety and confusion. Beyond merely stating the diagnosis, participants desired detailed explanations and guidance aligned with the patient’s level of understanding. Such communication may help reduce uncertainty and support women’s ongoing efforts toward biographical reconstruction by enabling them to better interpret their symptoms and engage more confidently in self-management.

*“When I received the diagnosis of PCOS via ultrasound, it would have been helpful to learn more about why it occurs, prevention strategies, or ways to alleviate it. Providing specific guidance would have been beneficial.”*

(Participant 13)

*“I wondered if I would have accepted treatment more readily if the explanation had been easier to understand… I think it would have helped if the doctor explained how to manage treatment or improve lifestyle habits.”*

(Participant 14)

#### 3.8.3. Demand for Patient-Tailored Materials

Participants noted a lack of accessible educational materials and content. They hoped for clear, easy-to-understand resources, case-sharing opportunities, and patient interaction platforms. Providing diverse and accessible educational tools—such as visual pamphlets and case examples—may help patients better understand their condition and identify practical ways to apply medical information in their daily lives.

*“It would be helpful if pamphlets were available in clinics, so that even without searching, patients could quickly grasp information about the condition. I also think doctors could provide simpler explanations from the patient’s perspective… Not using complicated medical terms, but materials that anyone can understand, and examples of milder cases available in the hospital would be beneficial.”*

(Participant 8)

*“I also thought it would be good to have opportunities to gather and talk with others who manage PCOS like a chronic condition or focus on quality of life.”*

(Participant 1)

## 4. Discussion

This study identified eight interrelated themes describing how Korean women make sense of and live with PCOS.

The first theme, ‘A Disease That Doesn’t Feel Like a Disease: Ambiguity and Reinterpretation’, captures the initial ambiguity surrounding the diagnosis. Participants reported experiencing considerable shock upon receiving a PCOS diagnosis. Some described the experience as “like being told I had cancer,” characterizing the diagnostic process as overwhelming and stating, “It kind of blew my mind that I could have all of these health problems down the line” [27]. This initial shock, which can be seen as the first stage of ‘disruption’ described by Bury, where existing expectations of life are shaken, imposes a psychological burden due to its chronic nature and potential impact on fertility, particularly for individuals already managing other health conditions, who may perceive PCOS as an additional and daunting diagnosis [11]. To alleviate initial confusion and anxiety, it is essential that healthcare providers offer clear explanations of the condition, its prognosis, and self-management possibilities, along with emotional support. Previous studies have predominantly approached PCOS symptoms, such as ovulatory dysfunction or amenorrhea, from a disease-centered perspective, framing them as sources of anxiety [28] or problems [29]. In contrast, some participants in this study reported reinterpreting the absence of menstruation as a convenience. This coping strategy appears when disease awareness is low or when participants emotionally accept the condition while finding their own way to manage societal expectations, reflecting an emotional aspect often overlooked in prior research [11,27]. Therefore, from the early stages of diagnosis, it is crucial to foster an accurate understanding of PCOS and provide educational approaches tailored to individual experiences.

In the second theme, ‘Isolation of Body and Mind Amid Stigma and Misunderstanding’, participants expressed concern over negative perceptions and stigma from others due to PCOS being a condition related to reproductive function, and they experienced significant emotional discomfort even when visiting gynecology clinics. This discomfort extended beyond clinical encounters, reflecting a sense of inadequacy arising from not meeting societal expectations of femininity and women’s roles [30]. The social stigma surrounding PCOS stems from misconceptions that it is merely a reproductive issue or the result of personal lifestyle choices [31]. In societies with strong social expectations regarding femininity and fertility, this stigma can exacerbate emotional distress, feelings of inadequacy, and social isolation among women with PCOS [32]. This sense of isolation reflects the social dimension of biographical disruption, in which illness not only reshapes bodily experiences but also challenges culturally prescribed identities and strains social connections. Thus, PCOS represents a complex experience that intersects reproductive health, self-identity, and societal norms of womanhood. Wright et al. [30] reported that PCOS acts as a “stigmatized disease,” producing both physical and emotional sequelae, with women perceiving themselves as victims of cultural stereotypes of femininity. Cultural differences in perceptions of fertility, body image, and gender roles mean that even women with the same diagnosis may experience varied forms of stigma [33]. In Western countries, reproductive health is regarded as an individual right, and open, inclusive discourses promote gender equality and social welfare, regardless of marital or childbearing status [34]. In contrast, Korea’s low birth rate policies tend to prioritize population control and childbirth promotion [35], which may intensify stigma and limit women’s access to holistic reproductive and overall health care, highlighting the need for system-level reforms such as establishing standardized PCOS care guidelines and integrating mental health screening into routine management. This underscores the need to shift the discourse in Korean society from childbirth-centered to health-rights-centered approaches. While clinical care has traditionally focused on physical symptoms of PCOS, its psychosocial impact is a critical aspect affecting women’s overall quality of life [36]. Women with PCOS must navigate social misunderstandings and stigma while managing the complex interplay between physical and mental health. Therefore, healthcare providers should adopt a multidimensional understanding of patients’ experiences, supporting disease acceptance and proactive coping strategies. Simultaneously, broader efforts are needed to promote accurate social awareness and policy approaches to recognize and support the health rights of women with PCOS.

The third theme, ‘Daily Life Limitation Caused by Visible Symptoms’, reveals that participants reported that the physical symptoms of PCOS, such as hirsutism, skin changes, and menstrual irregularities, caused not only practical discomfort in daily life but also emotional distress. One participant expressed, “I can’t even look in the mirror without wanting to cry”, revealing deep psychological pain related to appearance. These experiences align with previous research reporting that women with PCOS feel shame and isolation due to ‘unfeminine’ physiological symptoms [30]. In Korean society, strong sociocultural norms emphasize women’s appearance [37], making PCOS-related changes such as weight gain, hirsutism, or skin problems sources of social stigma. Irregular menstruation is also culturally significant, traditionally seen as an indicator of reproductive health [38], while abnormalities may be interpreted as dysfunction or illness [39]. These cultural expectations heighten anxiety, self-consciousness, and emotional distress in daily life. Therefore, the management of PCOS-related physical symptoms requires an approach that goes beyond medical treatment, incorporating psychosocial support and cultural sensitivity. To achieve this, it is crucial to provide education on lifestyle management strategies for irregular menstruation, non-stigmatizing information, and social support that enables patients to cultivate self-acceptance amidst unpredictable physical changes and appearance-related pressures [10,11]. Furthermore, there is a need to critically reflect on appearance-focused cultural norms and reproduction-centered concepts of femininity, while establishing social discourses and supportive systems where women with PCOS can safely share their experiences.

The fourth theme, ‘Ambivalent Feelings Surrounding Pregnancy’, reveals that participants experienced complex emotional distress as they navigated between the possibilities of pregnancy and infertility due to irregular menstruation and ovulation associated with their PCOS diagnosis. Some expressed fear of unplanned pregnancies caused by irregular ovulation, while at the same time feeling anxious about long-term infertility. This duality reflects a form of biographical disruption in which uncertainty about reproductive capacity challenges women’s anticipated life trajectory and their internalized sense of womanhood. Previous studies have reported that 76.1% of women with PCOS worried about not being able to have children in the future [40]. From the time of diagnosis, uncertainty about fertility has been identified as a significant source of psychological stress [41]. This anxiety can be further intensified in traditional, reproduction-centered societies [42]. In Korean society, shaped by Confucian cultural values, infertility is not only understood as a challenge to personal developmental tasks but also perceived as a threat to family lineage and continuity [43]. Consequently, uncertainty surrounding pregnancy extends beyond a medical or reproductive issue to deeply affect women’s identity and overall life planning. Healthcare providers must, therefore, understand the multilayered emotional complexity experienced by women with PCOS. Supportive counseling that acknowledges both the psychological impact of uncertainty and the cultural context in which women make meaning of their condition is essential, helping facilitate disease acceptance and the reconstruction of identity.

The fifth theme of this study, ‘Difficulties in Self-Management Due to Lack of Information’, highlights participants’ feelings of disappointment and confusion resulting from insufficient or fragmented explanations from healthcare providers about PCOS. Similar findings were reported by Soucie et al. [44], indicating that such experiences can weaken trust between patients and healthcare providers, leading patients to underestimate the seriousness of their condition or make unnecessary self-directed decisions, which, in turn, negatively affect treatment participation and self-management. When reliable information is lacking, patients tend to seek answers through the internet or peer networks [11], which can lead to the acquisition of inaccurate information. Therefore, it is important for healthcare providers to deliver clear and individualized information tailored to the patient’s level of understanding, along with empathetic communication, to support patients in becoming active participants in their treatment process. Although PCOS is diagnosed based on the Rotterdam criteria [21], patients often experience diagnostic ambiguity due to the condition’s diverse presentations and its overlap with other disorders [14], which can contribute to both underdiagnosis and overdiagnosis [45]. Such uncertainty is exacerbated by variations in clinical practice and the limited evidence regarding the long-term implications of PCOS [46]. These challenges highlight the importance of understanding patients’ perceptions and maintaining trust in healthcare providers throughout the diagnostic process to improve the quality of care [47]. Women with PCOS perceived that healthcare providers lacked sufficient knowledge about the condition [48] or were indifferent to their concerns [29]. As a result, they felt they did not receive adequate or reliable information, which negatively affected their motivation for self-care and healthy behaviors [48]. Participants in this study also emphasized the importance of early information provision, with some stating, “If I had understood the seriousness of this condition earlier, I would have tried harder.” This is consistent with previous findings showing that early intervention promotes positive health behavior changes [49]. Additionally, some participants reported that healthcare providers minimized the importance of PCOS or focused excessively on fertility-related issues, which caused emotional distress. For example, Authier et al. [50] reported that some women were told by their physicians, “Bit of a hormonal imbalance, come back and see me if you ever have trouble having children.” Similarly, Soucie et al. [44] found that when PCOS patients requested diagnostic tests or additional evaluations, they sometimes felt their requests were perceived by healthcare providers as “disgruntled” or “offensive.” These findings underscore the importance of healthcare providers offering individualized, clear information and empathetic communication to help patients become active participants in their care process. Providing accurate information at the initial diagnostic stage is a key factor in promoting disease acceptance and encouraging positive health behavior changes, forming the foundation for effective self-management and long-term disease control.

The sixth theme, ‘Psychological Exhaustion from Chronicity and Lack of Control’, describes how participants experienced frustration, helplessness, and resignation when symptoms showed little or no improvement despite prolonged management efforts. This experience is similar to “treatment fatigue” reported among individuals with other chronic illnesses [51], where the burden of long-term treatment leads to decreased adherence, discontinuation of health behaviors, and reduced self-efficacy [52]. PCOS requires consistent lifestyle and symptom management, yet improvements are often gradual or difficult to perceive, reinforcing the belief that “no matter how hard I try, nothing changes.” This sense of diminished control reflects the broader concept of chronic illness burden, in which individuals experience emotional exhaustion when personal effort does not appear to translate into meaningful symptom improvement. This perception of ineffectiveness is shaped not only by psychological reactions but also by interconnected factors, including sustained treatment demands, social stigma, and insufficient information. Therefore, interventions should emphasize both emotional regulation and restoration of perceived controllability. Evidence-based approaches such as mindfulness-based cognitive therapy (MBCT), mindfulness-based stress reduction (MBSR), and acceptance and commitment therapy (ACT) have been shown to reduce fatigue and emotional distress while improving quality of life in individuals with chronic illnesses [53,54]. Additionally, pacing strategies—effective in managing chronic fatigue—may help women with PCOS maintain a sustainable level of effort and avoid the cycle of overexertion followed by emotional depletion [55].

The seventh theme, ‘Awareness and Practices for Living with the Condition’, describes how, over time, participants gradually accepted PCOS and actively managed it through lifestyle modifications, information seeking, and engaging with family members. Family support emerged as a crucial resource for maintaining long-term motivation. Wright et al. [56] reported that women with PCOS develop and maintain self-management strategies as they progress through different life stages. Holbrey and Coulson [57] highlighted that participation in online support groups provides emotional support, access to information, and opportunities for shared decision making, enhancing self-management. Hadjiconstantinou et al. [41] demonstrated that group-based education positively influences disease understanding, emotional regulation, and self-care strategies. These findings indicate that effective PCOS management goes beyond individual efforts and benefits from combining social support with structured educational interventions. In Korea, interventions should consider the family-centered culture and societal emphasis on marriage and childbirth. Future research should track self-management across life stages and develop stage-specific support strategies.

The final theme, ‘Moving Toward a Patient-Centered Healthcare Environment’, highlights participants’ concerns about insufficient information, poor communication, and the lack of educational systems throughout the management of PCOS. They emphasized the need for early detection, preventive education, patient-friendly explanations, tailored educational materials, and opportunities for peer interaction. Presswala and De Souza [11] pointed out that inadequate information and a lack of empathy during the diagnostic process undermine patients’ trust in healthcare providers, underscoring the importance of patient-centered communication and shared decision-making. Ismayilova and Yaya [10] emphasized the need for age-specific support programs and expanded mental health resources, while Copp et al. [45] proposed phenotype-based counseling and multidisciplinary integrated care systems. Furthermore, Melson et al. [58] reported that ongoing counseling and integrated care approaches are key strategies for enhancing patient satisfaction and treatment adherence. These studies support the notion that participants’ expressed needs represent practical directions for healthcare improvement rather than mere personal desires. Thus, PCOS should be approached not only as a reproductive issue but also as a matter of overall health rights, encompassing metabolic and mental health. To achieve this, it is essential to strengthen comprehensive health education spanning adolescence through adulthood, provide clear, accessible educational materials and real patient case examples, create platforms for peer interaction and shared experiences, and establish tailored counseling services and long-term management systems. Such changes are expected to improve patients’ quality of life, prevent complications, and enhance the sustainability of disease management.

Across these themes, a core pattern emerged: Korean women with PCOS navigate their condition while managing sociocultural expectations around femininity and fertility. After diagnosis, many experienced confusion and disruption to their daily lives and self-perception, but gradually sought ways to understand and manage PCOS using both medical information and culturally shaped beliefs. As they adapted, PCOS came to be viewed as a chronic condition requiring ongoing management, supported by coping strategies such as lifestyle adjustments and redefining self-worth beyond reproductive ability. In line with Bury’s concept of biographical disruption [20], these findings show that living with PCOS involves negotiating identity and social expectations, rather than a simple progression toward acceptance.

### 4.1. Implications for Practice and Policy

The findings of this study highlight the need for patient-centered PCOS care that addresses not only physical symptoms but also the psychological and sociocultural challenges women encounter. Healthcare providers should offer clear explanations about diagnosis and long-term management, validate patients’ emotional distress, and provide consistent information to reduce confusion. Developing accessible educational materials—such as visual guides or mobile-based resources—may improve understanding and self-management. At a policy level, public awareness efforts and earlier menstrual health education in schools could help reduce stigma, promote early recognition of symptoms, and support equitable access to care for women with PCOS.

### 4.2. Limitation

This study has several limitations. First, participants were recruited from a fertility-focused clinical setting, which may have emphasized reproductive concerns over other aspects of PCOS. Second, all participants were relatively young Korean women, which limits the transferability of the findings to women of different ages, cultural backgrounds, or socioeconomic contexts. Third, social desirability bias may have influenced participants’ responses, as they might have underreported difficulties with lifestyle management or over-reported acceptance of their condition.

### 4.3. Recommendations for Future Research

Future research should examine how illness experiences differ across PCOS phenotypes, life stages, and sociocultural backgrounds to improve the tailoring of clinical interventions. Longitudinal or mixed-methods studies could further clarify how women’s perceptions, coping strategies, and healthcare needs evolve over time. Additionally, intervention-based research is needed to evaluate the effectiveness of patient-centered educational programs and psychosocial support models in improving mental health, treatment adherence, and overall quality of life.

## 5. Conclusions

This study provides an in-depth understanding of how Korean women experience and make sense of PCOS within a social and cultural context shaped by Confucian values and gendered expectations. Participants’ narratives reveal the tension between medical understandings of illness and deeply rooted beliefs about womanhood and fertility. These insights underscore that effective care for women with PCOS requires more than biomedical management; it demands empathy, open dialogue, and culturally sensitive care.

The findings highlight the importance of integrating social context into patient education and public health programs, ensuring that women receive information and care that affirm both their health and their sense of self. Overall, this research will contribute to ongoing conversations about reproductive health, gender equity, and the need for inclusive, compassionate healthcare, providing evidence to inform approaches that better reflect women’s lived experiences of PCOS. Together, these findings illustrate how PCOS functions as a culturally shaped biographical disruption for Korean women and emphasize the need for healthcare systems that actively support women’s biographical reconstruction across the life course.

## Figures and Tables

**Table 1:** Example of the analytic process from meaning units to codes and themes.

Meaning Unit (Participant Quote)	Condensed Meaning	Code	Category (Sub-Themes)	Theme
“I was so shocked, it felt like being given a cancer diagnosis.”	Diagnosis felt severe and frightening	Shock at diagnosis	Shock and confusion at diagnosis	A Disease That Doesn’t Feel Like a Disease: Ambiguity and Reinterpretation
“Many people view visiting a gynecologist negatively, and going alone is also embarrassing…”	Visiting a gynecologist feels stigmatizing	Gynecology visit stigma	Emotional discomfort during gynecological visits amid social perceptions	Isolation of Body and Mind Amid Stigma and Misunderstanding
“Even in midsummer, breakthrough bleeding kept happening… I never knew when my period would come.”	Unpredictable menstruation causes daily stress	Anxiety from unpredictable cycles	Anxiety and lifestyle limitations due to unpredictable menstruation	Daily Life Limitations Caused by Visible Symptoms

**Table 2:** Participants of this study.

ID	Age (Years)	Age at Diagnosis (Years)	Duration Since Diagnosis (Years)	Married (Yes/No)	Children (Yes/No)
1	23	19	4	No	No
2	26	19	7	Yes	No
3	34	18	9	Yes	No
4	33	27	8	Yes	No
5	30	23	7	Yes	No
6	36	29	7	Yes	Yes
7	32	27	5	Yes	No
8	35	19	16	Yes	No
9	36	25	11	Yes	Yes
10	37	36	1	Yes	No
11	31	23	8	Yes	No
12	36	29	7	Yes	No
13	31	21	10	Yes	No
14	35	19	16	Yes	No
15	28	17	11	No	No
M ± SD	32.2 ± 4.09	23.4 ± 5.37	8.5 ± 4.02		

**Table 3:** Themes of disease experience in women with PCOS.

Theoretical Framework	Theme	Sub-Theme
Disruption	A Disease That Doesn’t Feel Like a Disease: Ambiguity and Reinterpretation	Shock and Confusion at Diagnosis An Invisible Disease, Unfelt Risk Reinterpretation as Convenience
	Isolation of Body and Mind Amid Stigma and Misunderstanding	Social Perceptions That Discourage Disclosure Emotional Discomfort During Gynecological Visits Amid Social Perceptions Feelings of Being Alone Within the Disease
	Daily Limitations Caused by Visible Symptoms	Confusion and Embarrassment Due to Appearance Changes Anxiety and Lifestyle Limitations Due to Unpredictable Menstruation
	Ambivalent Feelings Surrounding Pregnancy	Anxiety About Unpredictable Fertility Fear of Infertility
Meaning making and coping work	Difficulties in Self-Management Due to Lack of Information	Patient Burden in Decision Making Due to Ambiguous Explanations for Healthcare Providers Negative Emotional Experience Due to Healthcare Providers’ Response to Symptoms
	Psychological Exhaustion from Chronicity and Lack of Control	Frustration and Helplessness During Ongoing Management Resignation and Surrender When Efforts Yield No Change Self-Blame from Belated Awareness
Biographical reconstruction	Awareness and Practices for Living with the Condition	Self-Acceptance Redefining the Conditions Through Awareness of Complications Proactive Information-Seeking for Condition Understanding Lifestyle Changes and Family Support
	Moving Toward a Patient-Centered Healthcare Environment	Need for Education and Awareness Improvement Expectation for Healthcare Providers’ Explanation, Empathy, and Guidance Demand for Patient-Tailored Materials

## Data Availability

The in-depth interview data used in this study contain sensitive personal information and carry a risk of participant identification; therefore, the complete raw data cannot be disclosed or shared. To ensure the credibility of this study, anonymized interview excerpts and a transparent description of the analytic process are provided within this article.

## Abbreviation

The following abbreviation was used in this manuscript:

PCOS	Polycystic ovary syndrome
